# Capsaicin decreases fecundity in the Asian malaria vector *Anopheles stephensi* by inhibiting the target of rapamycin signaling pathway

**DOI:** 10.1186/s13071-022-05593-0

**Published:** 2022-12-12

**Authors:** Jing Wang, Shasha Yu, Luhan Wang, Tingting Liu, Xuesen Yang, Xiaobing Hu, Ying Wang

**Affiliations:** 1 Department of Tropical Medicine, College of Military Preventive Medicine, Army Medical University, No. 30 Gaotanyan St, Shapingba Dis, Chongqing, 400038 China; 2 Centers for Disease Control and Prevention of Western Theater Command, Lanzhou, 730020 China

**Keywords:** Capsaicin, *Anopheles stephensi*, Fecundity, Vitellogenin, TOR signaling pathway

## Abstract

**Background:**

Mosquito-borne diseases threaten human health, but mosquito control faces various challenges, such as resistance to chemical insecticides. Thus, there is an urgent need for more effective and environment-friendly control agents. Capsaicin can downregulate the mTOR signaling pathway of tumor cells. The TOR signaling pathway can mediate the expression of vitellogenin (Vg) to regulate the fecundity of insects. Whether capsaicin has the potential to inhibit fecundity of mosquitoes by regulating TOR pathway and Vg expression is currently unclear.

**Methods:**

*Anopheles stephensi* were fed with blood of mice administered capsaicin by gavage or sugar containing capsaicin followed by a blood feeding with normal mice. Then, the engorged female mosquitoes were tubed individually and underwent oviposition. The eggs and individuals in the subsequent development stages, including larvae, pupae, and emerging adults, were counted and compared between the capsaicin treatment and control groups. Additionally, total RNA and protein were extracted from the engorged mosquitoes at 24 h post blood feeding. Real-time PCR and western blot were performed to detect the transcriptional level and protein expression of the key fecundity-related molecules of mosquitoes. Finally, TOR signaling pathway was inhibited via rapamycin treatment, and changes in fecundity and the key molecule transcription and protein expression levels were examined to verify the role of TOR signaling pathway in the effect of capsaicin on mosquito fecundity.

**Results:**

The laid and total eggs (laid eggs plus retained eggs) of *An. stephensi* were significantly reduced by feeding on the blood of capsaicin-treated mice (*P* < 0.01) or capsaicin-containing sugar (*P* < 0.01) compared with those in the control group. Moreover, the transcription and protein expression or phosphorylation levels of fecundity-related molecules, such as Akt, TOR, S6K, and Vg, were significantly decreased by capsaicin treatment. However, the effects disappeared between control group and CAP group after the TOR signaling pathway was inhibited by rapamycin.

**Conclusions:**

Capsaicin can decrease the fecundity of *An. stephensi* by inhibiting the TOR signaling pathway. These data can help us to not only understand the effect of capsaicin on the reproductive ability of *An. stephensi* and its underlying mechanism, but also develop new efficient, safe, and pollution-free mosquito vector control agents.

**Graphical Abstract:**

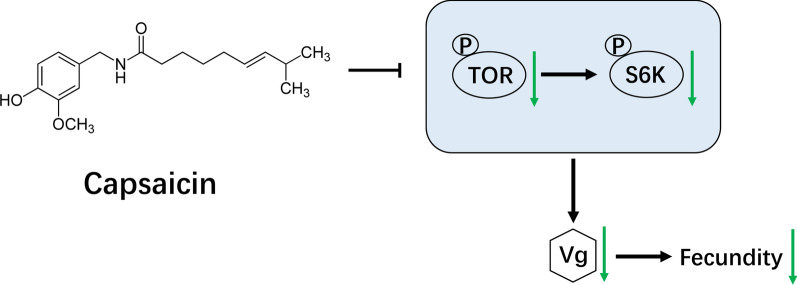

**Supplementary Information:**

The online version contains supplementary material available at 10.1186/s13071-022-05593-0.

## Background

Mosquitoes are responsible for more human deaths than any other animal, and around 725,000 people die from mosquito bites and mosquito-borne diseases each year [1, 2]. Mosquitoes can transmit various infectious diseases such as malaria, dengue fever, Zika, yellow fever, Japanese encephalitis, filariasis, and West Nile fever. Among these, malaria is one of the most devastating diseases worldwide, especially in poverty areas such as Africa. According to the WHO, approximately 241 million new cases and 627,000 deaths related to malaria were reported worldwide in 2020 [3]. More than 480 *Anopheles* species have been discovered, of which about 70 have been implicated in the transmission of human malaria, and approximately 40 are considered the main vectors of malaria. *Anopheles stephensi* originated in Southeast Asia and the Arabian Peninsula and rapidly expanded its geographical range to the Lakshadweep Islands (2001), the Horn of Africa (2012), Sri Lanka (2017), and the Republic of Sudan (2019). It has recently emerged as an efficient and invasive urban malaria vector [4].

Vector control is the most effective way to reduce the prevalence of mosquito-borne diseases. Chemical insecticides have been extensively and intensively used for several decades to control mosquitoes. However, this approach is hindered by increasing insecticide resistance, environmental pollution, and toxicity to humans. Therefore, there is an urgent need for more effective and environment-friendly alternatives.

Capsaicin (CAP) is a natural plant extract and the main irritant in chili peppers. CAP has a powerful effect on various physiological processes in animals [5]. CAP causes a burning sensation through chemoreceptors and nociceptors [6, 7]. Therefore, CAP causes irritation in numerous species, ranging from insects to mammals, and can be used as a repellent. CAP also affected the oviposition decisions of several insects and insect spawning and caused abnormal intestinal development [8, 9]. In addition, CAP induced the autophagy and apoptosis of tumor cells through endoplasmic reticulum stress and the downregulation of the AKT/mTOR pathway [10–14]. The target of rapamycin (TOR) signaling pathway plays a pivotal role in regulating insect reproduction by mediating the expression of vitellogenin (Vg) [15–18]. However, the underlying mechanisms are still unclear, and the effect of CAP on the reproductive capacity of *Anopheles* mosquitoes has not been reported yet.

In this study, we aimed to elucidate the effect of CAP on the reproductive ability of *An. stephensi*. The relationship between TOR signaling pathway-related mechanisms and this effect was also investigated. This study will be helpful in the development of new efficient, safe, and pollution-free mosquito vector control agents.

## Methods

### Mosquito rearing

The *An. stephensi* Hor strain was maintained at 28 °C and 70–80% relative humidity with a 12-h light/dark photocycle, according to the standard rearing procedures in the laboratory. The adults and larvae were fed on 10% sugar solution and a powdered mix of dry degreased pork liver and yeast at a ratio of 1:4, respectively [19].

### Capsaicin treatment

To investigate the impact of a CAP-treated blood meal on the fecundity of *An. stephensi*, normal Kunming mice (starved overnight) were administered 4 mg/kg CAP via gavage, and 3-day-old *An. stephensi* were allowed to feed on them 1.5 h later. To further confirm the direct effect of CAP on the fecundity of *An. stephensi*, 3-day-old adult mosquitoes were fed with 10% sugar solution (control group) or 10% sugar solution containing 50 μM CAP (treatment group). Two days later, normal blood feeding on mice was performed.

### Impact of capsaicin treatment on the fecundity of *An. stephensi*

After blood feeding, the engorged females were housed individually. Wet filter paper was provided to induce oviposition on day 3 post blood feeding and the laid eggs were counted. Ten days later, the ovaries were dissected and the retained eggs were counted. The laid eggs were placed in water, hatched to larvae, and reared to the fourth instar. Further development into pupae and adults was monitored. The hatch, pupation, and emergence rates were calculated based on these counts. The egg counts and gravidity, oviposition, hatching, pupation, and emergence rates were compared between the control and CAP-treated groups.

### Real-time PCR

At 24 h post blood feeding, 10 engorged mosquitoes from each group were anesthetized at −20 °C for 10 min and homogenized on ice. The total RNA was extracted using TRIzol reagent (Invitrogen, Carlsbad, CA, USA). Then, 1 μg of total RNA was reverse transcribed into cDNA using a reverse transcription kit (Takara, Dalian, Liaoning, China) as per the manufacturer’s protocol. Real-time fluorescent quantitative PCR was performed with a Bio-Rad CFX96 Touch™ real-time PCR instrument (Bio-Rad, Hercules, CA, USA) using the KAPA SYBR^®^ FAST qPCR kit (KAPA Biosystems, Wilmington, MA, USA) to detect the transcriptional level of the key fecundity-related genes of *An. stehpensi*, including *AsAKT*, *AsTOR*, *AsS6K*, and *AsVg*. The conserved *AsS7* of *An. stephensi* was used as the internal reference gene. The PCR reactions (20 μl) contained 0.8 μl of each primer (10 μM), 10 μl KAPA SYBR® FAST qPCR Kit Master Mix 2 × Universal (KAPA Biosystems, Wilmington, MA, USA), 7.9 μl ddH_2_O, and 0.5 μl cDNA. The SYBR Green quantitative PCR cycling conditions consisted of initial denaturation at 95 °C for 3 min, followed by 40 amplification cycles of 3 s at 95 °C and 30 s at 60 °C, and a melt curve step from 65 °C to 95 °C with increment of 0.5 °C every 5 s. The expression of each gene relative to the ribosomal *AsS7* RNA was determined using the 2^−ΔΔCT^ method. The primers are listed in Additional file 1: Table S1.

### Western blot

Twenty engorged mosquitoes from each group were anesthetized at −20 °C 24 h after blood feeding and homogenized to extract the total protein using western blot and immunoprecipitation cell lysate (Beyotime, Shanghai, China). The proteins were denatured at 95 °C for 5 min, separated using a 10% SDS-PAGE gel, and then transferred to a PVDF membrane. After being blocked with 5% skimmed milk powder for 1 h, the membrane was incubated with primary antibodies against the key fecundity-related molecules of mosquitoes, including Vg, phosphorylated AKT (p-AKT), TOR (p-TOR), TOR substrate ribosomal S6 kinase (p-S6K), whose phosphorylation level can be used as an indicator of TOR activity [20–22], and the reference protein β-actin at 4 °C overnight. Subsequently, the membrane was washed with TBS-T solution three times and incubated with the secondary antibody for 1 h at room temperature. After being washed again, the membrane was finally visualized (ChemiDOC^TM^MP Imaging System, BIO-RAD). ImageJ software was used to quantify the signal intensity.

### Rapamycin pretreatment to confirm the role of the TOR signaling pathway in the impact of CAP on *An. stephensi* fecundity

Rapamycin (RAPA) can specifically inhibit the function of TOR. To further confirm the role of the TOR signaling pathway in the impact of CAP on *An. stephensi* fecundity, 1-day-old adult mosquitoes were fed on 10% sugar solution containing 20 μM RAPA, while the control group was fed on 10% sugar solution without RAPA. Two days later, Kunming mice administered with CAP via gavage and normal mice were used for blood feeding the mosquitoes in the RAPA and control groups, respectively. After 24 h, protein was extracted from the engorged female mosquitoes. Subsequently, the protein levels of p-TOR and Vg were detected using western blot method. The remaining engorged females were separated and reared individually. The number of eggs laid by each female was counted and recorded.

### Statistical analysis

Chi-square test was used to compare the gravidity, oviposition, hatching, pupation, and emergence rates. The Mann-Whitney rank sum test was used for the abnormally distributed data to compare the egg counts of the CAP-treated group and control group, and t-tests were used to compare the normally distributed data. All statistical analyses were performed using the GraphPad Prism software (version 8) and SigmaStat software (version 3.5).

## Results

### Blood feeding on mice administered CAP decreased *An. stephensi* fecundity

**Fig. 1:**
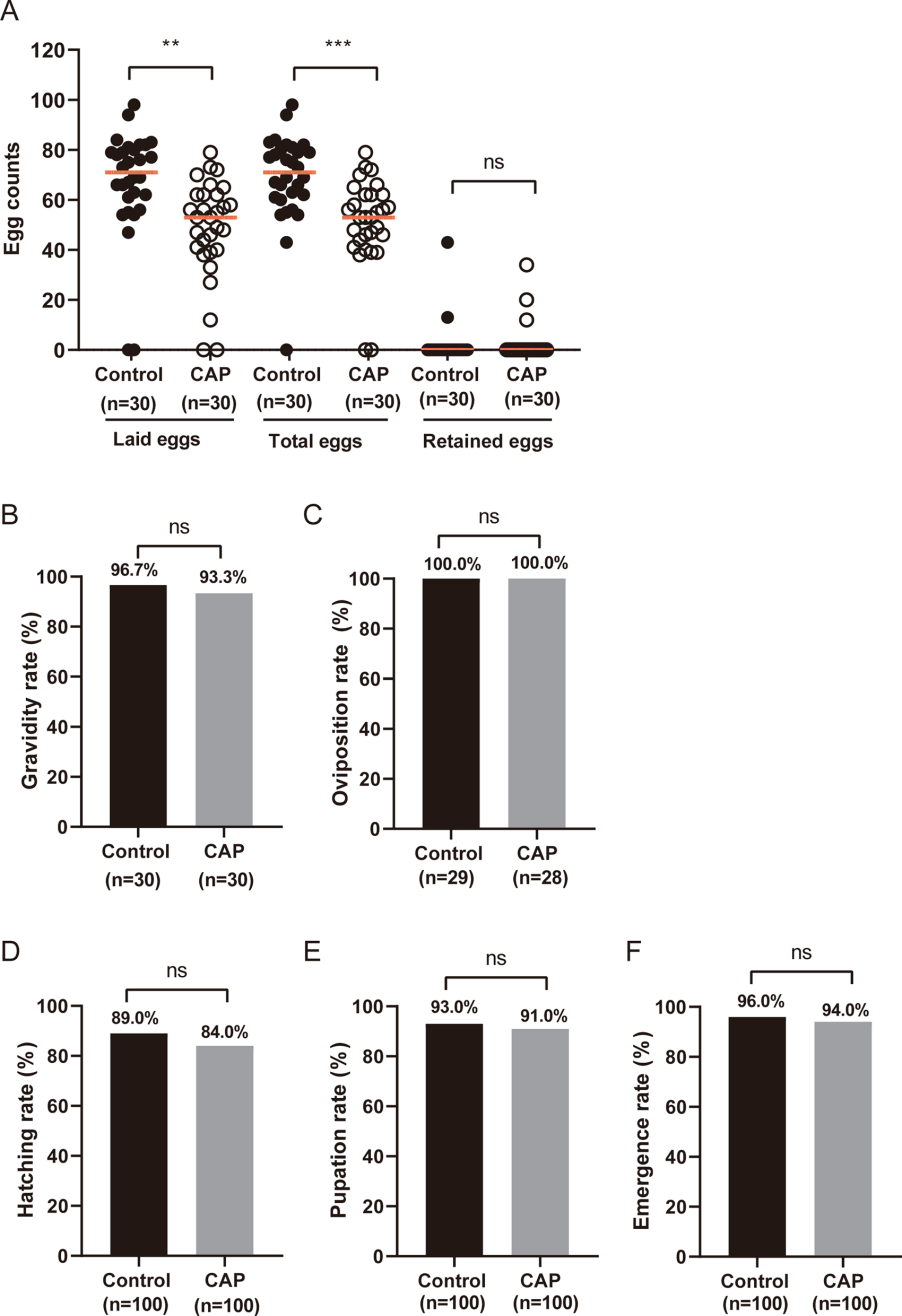
The fecundity of *Anopheles stephensi* was significantly decreased by blood feeding on CAP-treated mice. **A** The counts of laid, total, and retained eggs produced by female mosquitoes after blood feeding on normal mice and CAP-treated mice. The dots represent egg counts. The red horizontal line represents the median egg count. **B** The gravidity rates of mosquitoes in the control and CAP groups. **C** The oviposition rates of the mosquitoes in the control and CAP groups. **D** The hatching rates of the laid eggs from the control and CAP groups. **E** The pupation rates of the larvae from the control and CAP groups. **F** The emergence rates of the pupae from the control and CAP groups. **P < 0.01; ***P < 0.001; ns, no significant difference

To investigate the effect of CAP-treated blood on the fecundity and development of *An. stephensi*, we focused on four key steps: oviposition, hatching, pupation, and eclosion. The results showed that the laid and total egg (laid eggs plus retained eggs) counts of *An. stephensi* fed on the blood of CAP-treated mice were significantly lower than those of the control group fed on normal mice (*P* < 0.01). There was no significant difference in the retained egg counts or rates of gravidity, oviposition, hatching, pupation, or eclosion between the control and CAP groups (all *P* > 0.05) (Fig. 1). This indicates that CAP treatment could reduce egg production but has no impact on gravidity or oviposition rates or the subsequent development of *An. stephensi* after laying.

### CAP affects the transcription level and protein expression of fecundity-related molecules in *An. stephensi*

**Fig. 2:**
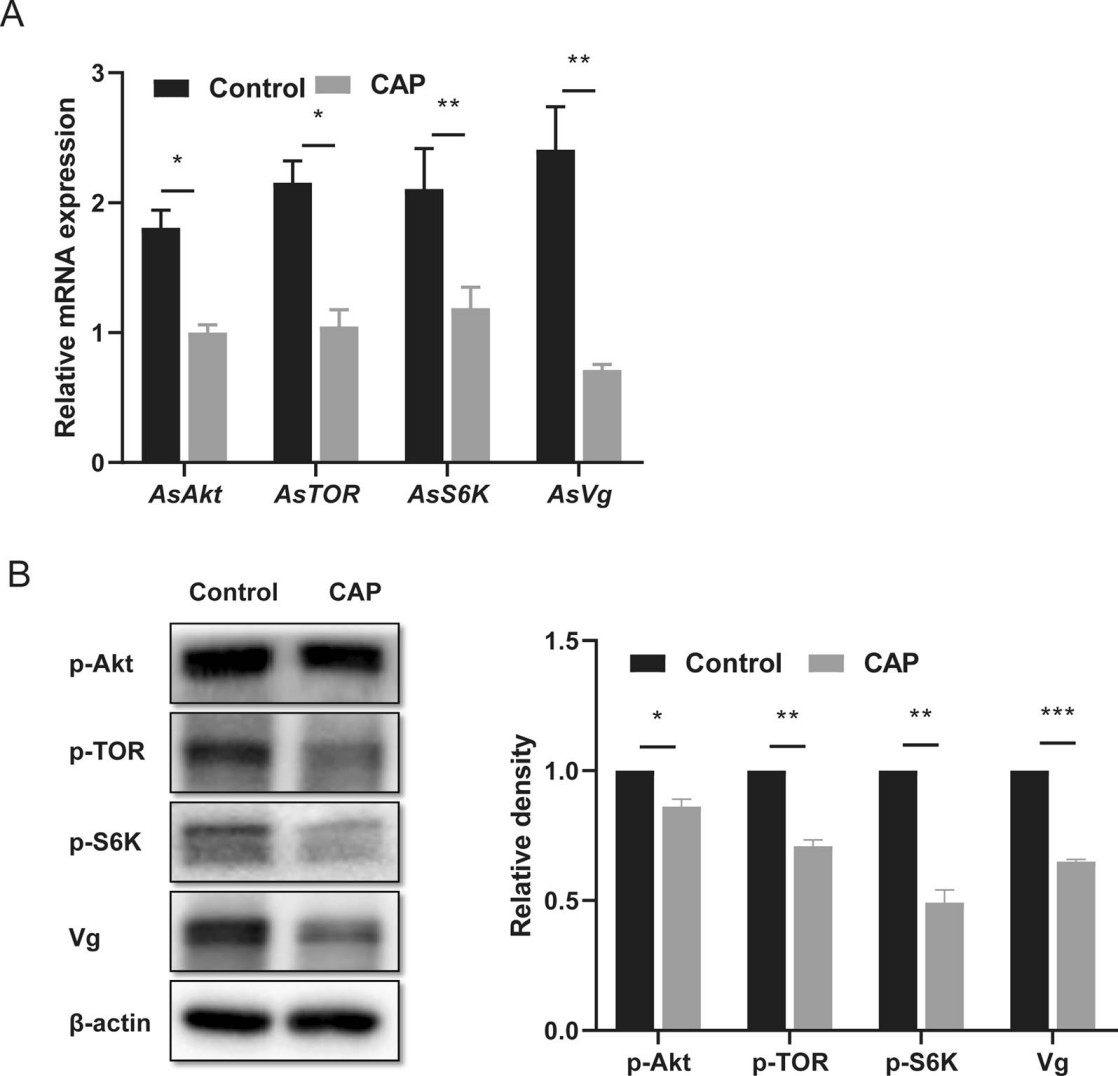
The TOR signaling pathway was significantly inhibited by blood feeding on CAP-treated mice. **A** The transcription levels of the key fecundity-related genes (*AsAkt*, *AsTOR*, *AsS6K*, and *AsVg*) were significantly lower in the CAP group than in the control group. **B** The western blot results showed that the phosphorylated AKT (p-AKT), TOR (p-TOR), and S6K (p-S6K) levels and Vg protein levels relative to the reference protein β-Actin were obviously downregulated by CAP pretreatment. This indicates that CAP inhibited the TOR signaling pathway. Data analysis was confirmed by *t*-test. *P < 0.05; **P < 0.01; ***P < 0.001; ns, no significant difference

To further investigate the molecular mechanisms underlying the effect of CAP on mosquito fecundity, real-time PCR and western blot were performed to detect changes in the transcription and protein levels of the key molecules in the TOR signaling pathway, respectively. Compared with that in the control group, CAP treatment significantly decreased the mRNA levels of the *AsAkt*, *AsTOR*, *AsS6K*, and *AsVg* genes (Fig. 2A). Data from three independent western blots were statistically analyzed. Compared with the control group, CAP pretreatment significantly reduced the protein phosphorylation or expression levels of p-AKT, p-TOR, p-S6K, and Vg (Fig. 2B). These results indicate that CAP can inhibit the transcription and protein expression or phosphorylation of the key molecules in the TOR signaling pathway of *An. stephensi*.

### The TOR signaling pathway was confirmed to be involved in the effect of CAP on the reproductive ability of *An. stephensi*

**Fig. 3:**
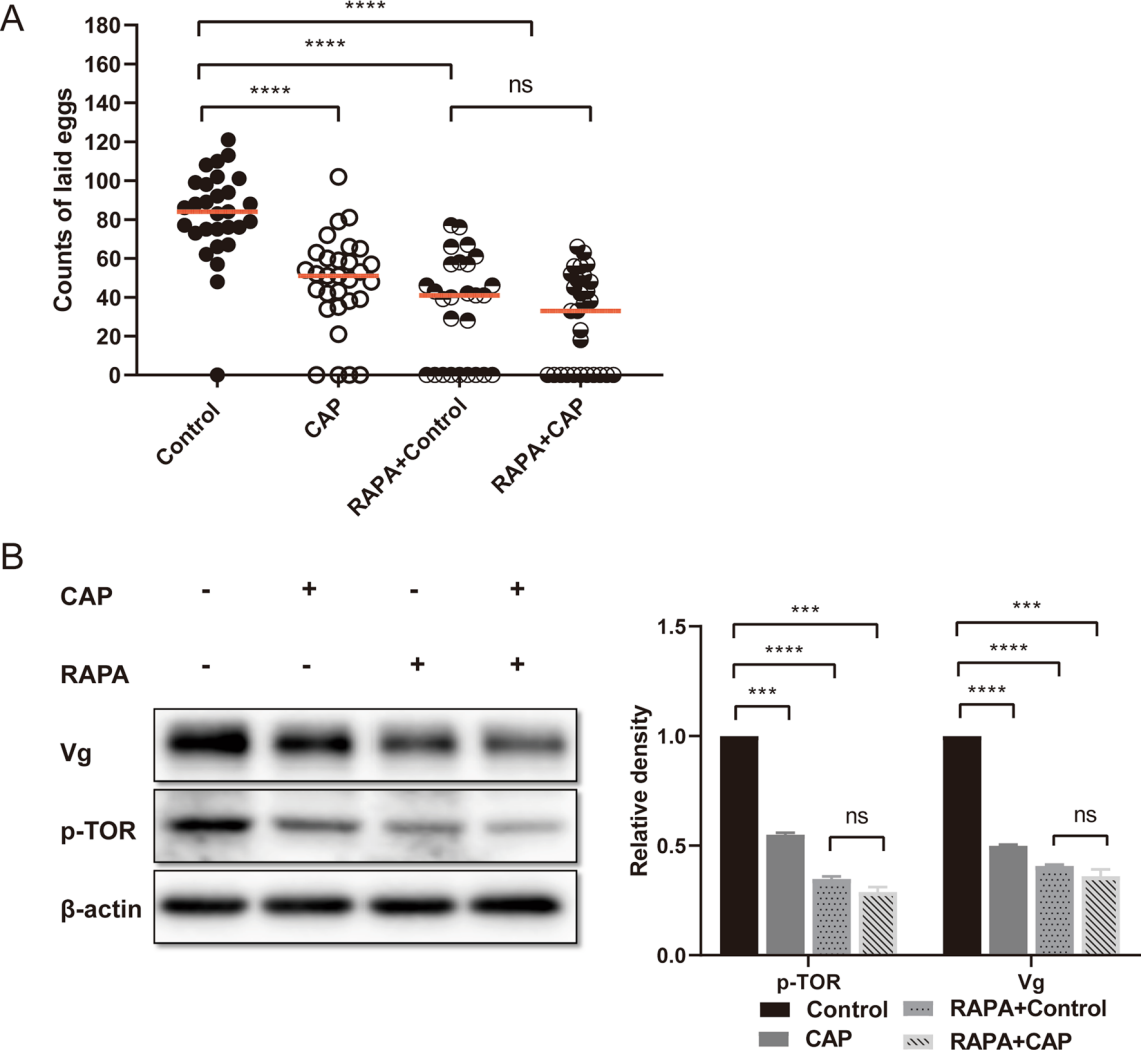
The role of the TOR signaling pathway was confirmed by RAPA pretreatment. **A** The comparisons of egg counts showed that significantly fewer eggs were laid in the CAP group than in the control group, but the difference disappeared under pretreatment with RAPA. **B** Western blot was conducted to detect the protein levels of p-TOR and Vg. Beta-actin was used as the internal reference protein. The phosphorylated TOR and Vg protein levels were obviously downregulated by CAP pretreatment, but the difference disappeared under pretreatment with RAPA. Data analysis was confirmed by *t*-test. ***P < 0.001, ****P < 0.0001; ns, no significant difference

Consistent with the previous results, the counts of laid eggs were significantly reduced by CAP compared to the normal blood feeding control group, without RAPA administration. However, the difference in egg counts between the control and CAP groups disappeared after pretreatment with the TOR inhibitor RAPA (Fig. 3A). The western blot results showed that CAP treatment significantly decreased the protein level of p-TOR and Vg without RAPA, but there was no significant difference between the two groups when treated with RAPA (Fig. 3B). This confirmed that CAP can reduce the reproductive ability of *An. stephensi* by inhibiting the TOR signaling pathway.

### CAP exerts a direct effect on *An. stephensi* fecundity

**Fig. 4:**
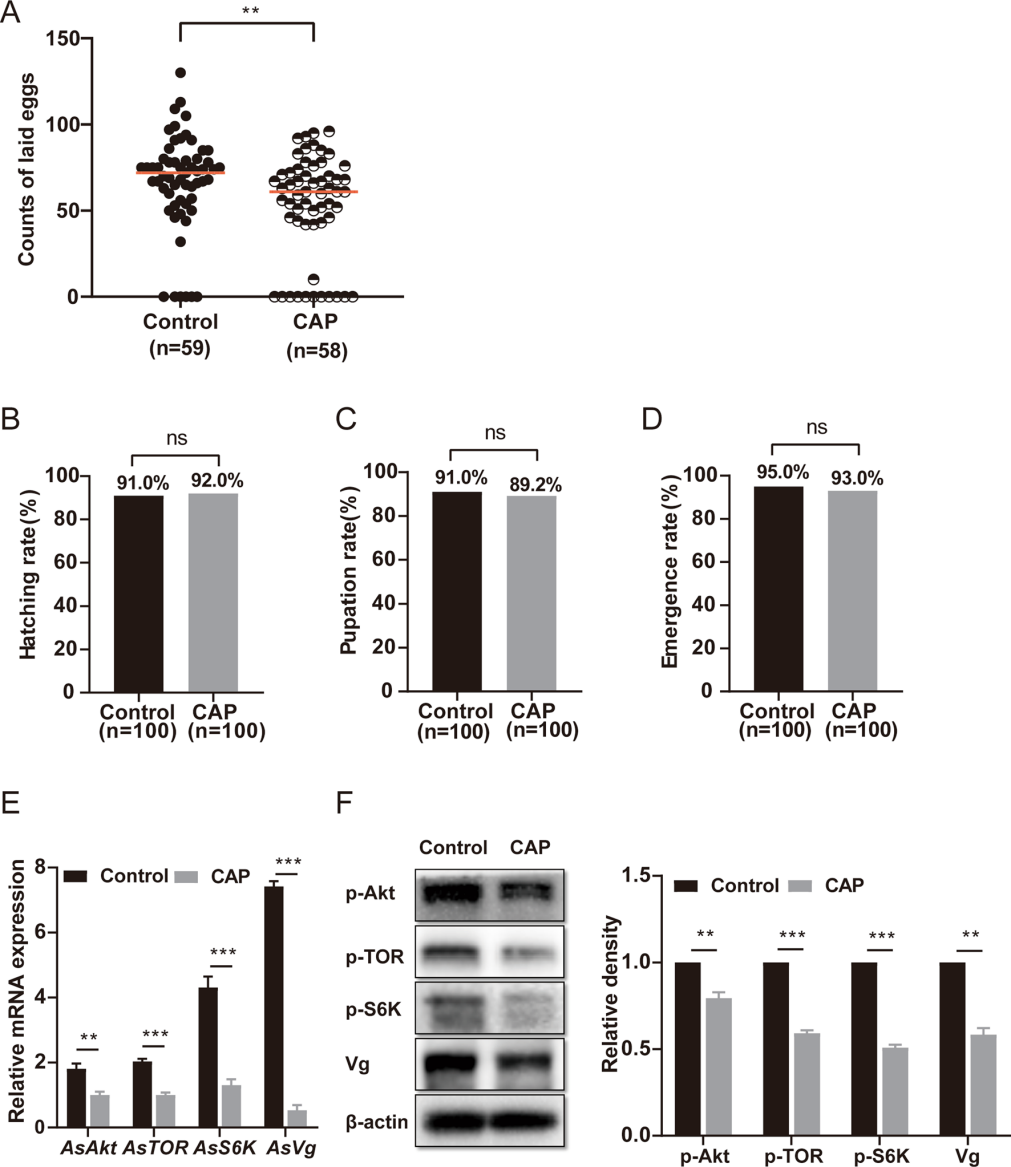
Direct feeding with CAP-containing sugar reduced the laid eggs and inhibited the TOR signaling pathway. **A** CAP-containing sugar solution feeding significantly reduced the number of eggs laid by female mosquitoes. The red horizontal line represents the median count of laid eggs. **B**, **C**, **D** Comparisons of the hatching, pupation, and emergence rates between the control and CAP groups. **E** Real-time PCR results showed that feeding adult mosquitoes with CAP-containing sugar solution significantly decreased the mRNA levels of the *AsAkt*, *AsTOR*, *AsS6K*, and *AsVg* genes compared with those in the control group. **F** The western blot results showed that the protein levels of p-AKT, p-TOR, p-S6K, and Vg relative to the reference protein β-actin were significantly lower in the CAP group than in the control group. Data analysis was confirmed by *t*-test. **P < 0.01; ***P < 0.001; ns, no significant difference

To understand the direct effect of CAP on mosquito fecundity, adult *An. stephensi* were fed with 10% sugar solution containing 50 μM CAP for 2 days. The control group was fed a sugar solution without CAP. Then, the mosquitoes were fed on the blood of normal mice. After 24 h, total RNA and protein were extracted from engorged female mosquitoes. Real-time PCR and western blot were performed to detect the mRNA and protein levels of key molecules. The remaining engorged females were reared individually, and the eggs laid by each mosquito were counted. The hatching, pupation, and emergence rates were also investigated, as before. As shown in Fig. 4A, the egg counts were significantly reduced by CAP feeding (*P* = 0.0077 < 0.01). There was no significant difference in the hatching, pupation, or emergence rates between the control and CAP groups (Fig. 4B, C and D). The transcription levels of the key fecundity-related genes (*AsAkt*, *AsTOR*, *AsS6K*, and *AsVg*) and protein expression or phosphorylation levels of Akt, TOR, S6K, and Vg were significantly decreased by CAP treatment according to the real-time PCR and western blot results (Fig. 4E and F). These results indicate that direct feeding with CAP can significantly reduce the reproductive ability of *An. stephensi* and inhibit the TOR signaling pathway.

## Discussion

Mosquito-borne diseases constitute one of the largest public health burdens faced by humanity. Mosquitoes are notorious as the most common and widely distributed vectors of infectious diseases worldwide. Mosquito control has always been a vital component of mosquito-borne disease control strategies [23]. Integrated control, including chemical insecticides, bio-control methods, physical methods, and gene manipulation methods, is recommended for the effective control of mosquitoes. The use of chemical insecticide is still the most common choice to control mosquitoes, especially in endemic areas. However, because of the well-known disadvantages of chemical insecticides, novel and more effective mosquito control strategies may be helpful.

Chili pepper is a favored spice in numerous regions. CAP is the main active ingredient in chili peppers. As an agonist of transient receptor potential vanilloid 1, CAP has wide application prospects in analgesic therapy, the prevention and treatment of cardiovascular and cerebrovascular diseases, anti-tumor treatments, and the treatment of immune-related diseases and obesity [24]. Moreover, increasing attention has been focused on the use of CAP in the control of pests and vector-borne diseases [25, 26]. When chili peppers are ingested by humans, CAP enters the human body, and 85%–95% of CAP can be rapidly absorbed through the gastrointestinal tract. The absorbed capsaicin is mainly metabolized by liver cytochrome P450, and a small part is hydrolyzed in the small intestine. The biological characteristics of mosquitoes, such as their reproductive ability, can thus be affected when they suck the blood of a person who has eaten chili peppers. To investigate this interesting issue, *An. stephensi* mosquitoes were fed on the blood of mice treated with CAP via gavage. Then, the fecundity of the mosquitoes was examined. The numbers of laid eggs and total eggs were significantly reduced by CAP pretreatment, although no difference was observed in the number of retained eggs between the control and CAP groups. This indicates that CAP can inhibit mosquito egg formation without affecting egg laying. Our previous study found that exposure to *Bacillus sphaericus* significantly reduced the oviposition ability of surviving *Anopheles dirus*, without affecting egg formation [27]. These results indicate that the effect and underlying mechanisms of CAP in relation to the fecundity of *An. stphensi* and of *Bacillus sphaericus* on the fecundity of *Anopheles dirus* are different.

Ingestion of blood is required for vector mosquitoes to initiate reproductive cycles determining their role as vectors of devastating human diseases. The TOR signaling pathway is one of the most crucial signals, which indicates the presence of blood in the midgut, then transduces the amino acid signal and finally activates the main vitellin precursor gene Vg [15, 18]. Vg synthesis and yolk production are important processes for mosquito development and reproduction [28–34]. As shown in our research, CAP can inhibit egg production in *An. stephensi*. Vitellogenesis is a prerequisite for insect egg laying and embryonic development. During insect vitellogenesis, Vg is mainly synthesized in fat body cells and transported by hemolymph through the intercellular spaces of follicular epithelial cells to reach the oocyte membrane. TOR, an evolutionarily conserved serine/threonine protein kinase, plays a pivotal role in mosquito vitellogenesis. A blood meal obtained by a female mosquito serves as a major source of amino acids and other nutrients for the upcoming reproductive cycle [35]. Amino acid feeding induces S6K phosphorylation mediated by TOR [36]. Amino acids are recognized and then transported into the fat body cells of the mosquito where they participate in the activation of TOR kinase via Ras homolog enriched in brain (Rheb) [37]. The activated TOR kinase phosphorylates downstream proteins and thereby derepresses ribosome biogenesis and protein translation via the inactivation of the translational inhibitor eukaryotic translation initiation factor 4E-binding protein and the activation of p70 S6 kinase (S6K) [22, 38]. Activated S6K elicits the massive translation of GATAa transcription factor, which replaces the repressive GATAr in the Vg gene promoter, thereby activating Vg transcription [18, 39]. Akt is a critical signaling molecule operating in multiple signaling pathways, including the insulin/insulin growth factor 1 signaling (IIS) and TOR cascades that regulate diverse physiologies in a wide range of eukaryotic organisms. In transgenic *Aedes aegypti* and *An. stephensi* females, the fat body-specific overexpression of active Akt increased Vg expression [40].

To understand the molecular mechanisms of the impact of CAP on mosquito fecundity, real-time PCR and western blot were performed to detect changes in the transcription and protein expression or phosphorylation levels of key fecundity-related molecules, such as Akt, TOR, S6K and Vg. The results show that CAP can inhibit the transcription and protein expression or phosphorylation of key molecules in the TOR signaling pathway of *An. stephensi*, suggesting that the TOR signaling pathway is involved in the impact of CAP on mosquito fecundity. To further confirm the role of the TOR signaling pathway on the impact of CAP on *An. stephensi* fecundity, the impact of CAP on egg production was investigated after inhibiting the TOR signaling pathway using RAPA. The differences in egg counts and protein levels of the key molecules between the CAP group and the control group disappeared under RAPA treatment. This suggests that CAP can decrease the fecundity of mosquitoes by inhibiting the TOR signaling pathway.

CAP can be rapidly absorbed in the stomach and small intestine of mice, and some of it is metabolized during the absorption process [41–43]. Although it has been demonstrated that feeding *An. stephensi* with the blood of CAP-treated mice can effectively inhibit their reproductive ability, it remains unclear whether this inhibitory effect is the result of CAP itself or its metabolites. To further understand whether the inhibitory effect is the result of CAP itself or its metabolites, the direct effects of CAP on mosquito fecundity and TOR signaling pathway status were also investigated. The results showed that directly feeding *An. stephensi* with a CAP-containing sugar solution could reduce their egg counts and inhibit the TOR signaling pathway. This indicates that CAP itself can directly decrease the fecundity of *Anopheles* mosquitoes by inhibiting the TOR signaling pathway. However, the possibility of CAP metabolites playing a role in impacting mosquito fecundity cannot be excluded. This study suggests that the disruption of the TOR signaling pathway of mosquitoes, for example by using CAP, may be an efficient vector control strategy. Moreover, CAP shows potential in reducing the population density of mosquitoes, indicating that chili peppers can prospectively be used in mosquito control.

There are still some interesting questions about the mechanism of the effect of capsaicin on mosquito fecundity. For example, does CAP circulate in hemolymph and enter the fat body thereby inhibiting TOR signaling? Ahn et al. [44] found that the fat body contained relatively higher enzyme activity of capsaicin glucoside than other tissues such as Malpighian tubules, testes, midgut and labial gland after *Helicoverpa armigera* were fed on capsaicin-supplemented artificial diet. This indicates that CAP might enter the fat body of insects and inhibit TOR signaling. Another interesting issue is whether autophagy is involved in the effect of CAP on mosquito fecundity. Several papers have suggested that capsaicin can induce autophagy. It has been shown that autophagy is inhibited by TOR under standard nutrition. So, CAP might activate autophagy of mosquitoes by inhibiting TOR according to our results. Upregulation of autophagy by rapamycin through inhibition of TOR mediates the lifespan extension of *Drosophila menanogaster*. Downregulation of autophagy blocks rapamycin-mediated life span extension. Consistent with our findings, the female fecundity was significantly reduced by inhibiting TOR activity after feeding rapamycin to *Drosophila menanogaster* [45]. In a different way, when autophagy was upregulated by feeding *Drosophila menanogaster* through dietary addition of Torin1, a well-established activator of autophagy via inhibition of the TOR pathway, the lifespan was extended and fertility was significantly elevated [46]. TOR kinase is a central component of two protein complexs: TORC1 and TORC2. Rapamycin is generally considered to be a specific inhibitor of TORC1. Unlike rapamycin, Torin1 is reported to inhibit kinase function in both TORC1 and TORC2 [47]. So, there may be diverse mechanisms of the different effects on fecundity of insects by rapamycin and Torin1. According to the existing literature report, there is a clear relationship among TOR, autophagy and lifespan. However, it is not yet clear whether autophagy is involved in fecundity of insects. Whatever the result, it is interesting to further explore the relationship of CAP, TOR, autophagy and fecundity.

This study focused on the impact of CAP on the fecundity of mosquitoes. Whether CAP can affect the vector competence of mosquitoes is another interesting issue. It has been reported that autophagy can promote or limit viral replication. In the case of the dengue fever virus, autophagy supports the viral replication cycle, and autophagic vesicles increase after infection [48]. Moreover, CAP can mediate cell autophagy [14, 49]. Studies have also pointed out that the silencing of AKT or TOR significantly reduces the dengue fever virus titer in mosquitoes and effectively inhibits the spread of the virus by mosquitoes [50]. As shown in this study, CAP can significantly inhibit the TOR signaling pathway in mosquitoes. The TOR pathway plays a key role in regulating the immune response of mosquitoes [51, 52]. The TOR pathway has an antagonistic relationship with immune response and regulates the immune response responsible for eliminating parasites. The inhibition of TOR activity can induce the expression of NF-κB transcription factor Rel2, which controls the synthesis of downstream anti-*Plasmodium* immune effectors. Inhibiting the TOR signaling pathway can indeed protect *An. stephensi* from *Plasmodium* infection [53]. Thus, our future study will focus on the potential of CAP in the transmission blocking of dengue fever and malaria.

## Conclusions

This study focused on the effect of CAP on the fecundity of *An. stephensi* and the underlying mechanisms. CAP pretreatment significantly reduced the number of laid eggs and total eggs and decreased the expression of key molecules in the TOR signaling pathway. When the TOR signaling pathway was specifically inhibited by RAPA, the effect of CAP on mosquito spawning disappeared. These results indicate that CAP can decrease the fecundity of *An. stephensi* by inhibiting the TOR signaling pathway. This study can help us to not only understand the effect and mechanism of CAP on the reproductive ability of *An. stephensi*, but also develop new efficient, safe and pollution-free mosquito vector control agents.

## Supplementary Information

**Additional file 1: Table S1**. The primers used in the real-time PCR.

## Data Availability

All data supporting the conclusions of this article are included within the article and Additional file 1. The datasets used and/or analyzed during the current study are available from the corresponding author upon reasonable request.

## Abbreviations

*An. stephensi*: *Anopheles stephensi*
CAP: Capsaicin
Vg: Vitellogenin
TOR: Target of rapamycin
RAPA: Rapamycin
S6K: S6 kinase

## References

[1] The Lancet Global Health (2017). Vector control: time for a planetary health approach. Lancet Glob Health.

[2] Abdellahoum Z, Nebbak A, Lafri I, Kaced A, Bouhenna MM, Bachari K (2022). Identification of Algerian field-caught mosquito vectors by MALDI-TOF MS. Vet Parasitol Reg Stud Reports.

[3] WHO (2021). World malaria report 2021.

[4] Ishtiaq F, Swain S, Kumar SS (2021). *Anopheles stephensi* (Asian Malaria Mosquito). Trends Parasitol.

[5] McCarty MF, DiNicolantonio JJ, O'Keefe JH (2015). Capsaicin may have important potential for promoting vascular and metabolic health. Open Heart.

[6] Latorre R, Brauchi S, Orta G, Zaelzer C, Vargas G (2007). ThermoTRP channels as modular proteins with allosteric gating. Cell Calcium.

[7] Al-Anzi B, Tracey WD, Benzer S (2006). Response of *Drosophila* to wasabi is mediated by painless, the fly homolog of mammalian TRPA1/ANKTM1. Curr Biol.

[8] Li Y, Bai P, Wei L, Kang R, Chen L, Zhang M (2020). Capsaicin functions as *Drosophila* ovipositional repellent and causes intestinal dysplasia. Sci Rep.

[9] Cowles RS, Keller JE, Miller JR (1989). Pungent spices, ground red pepper, and synthetic capsaicin as onion fly ovipositional deterrents. J Chem Ecol.

[10] Dai N, Ye R, He Q, Guo P, Chen H, Zhang Q (2018). Capsaicin and sorafenib combination treatment exerts synergistic antihepatocellular carcinoma activity by suppressing EGFR and PI3K/Akt/mTOR signaling. Oncol Rep.

[11] Hong ZF, Zhao WX, Yin ZY, Xie CR, Xu YP, Chi XQ (2015). Capsaicin enhances the drug sensitivity of cholangiocarcinoma through the inhibition of chemotherapeutic-induced autophagy. PLoS ONE.

[12] Ying H, Wang Z, Zhang Y, Yang TY, Ding ZH, Liu SY (2013). Capsaicin induces apoptosis in human osteosarcoma cells through AMPK-dependent and AMPK-independent signaling pathways. Mol Cell Biochem.

[13] Lin YT, Wang HC, Hsu YC, Cho CL, Yang MY, Chien CY (2017). Capsaicin induces autophagy and apoptosis in human nasopharyngeal carcinoma cells by downregulating the PI3K/AKT/mTOR Pathway. Int J Mol Sci.

[14] Qiao Y, Wang L, Hu T, Yin D, He H, He M (2021). Capsaicin protects cardiomyocytes against lipopolysaccharide-induced damage via 14-3-3gamma-mediated autophagy augmentation. Front Pharmacol.

[15] Zhai Y, Sun Z, Zhang J, Kang K, Chen J, Zhang W (2015). Activation of the TOR signalling pathway by glutamine regulates insect fecundity. Sci Rep.

[16] Maestro JL, Cobo J, Belles X (2009). Target of rapamycin (TOR) mediates the transduction of nutritional signals into juvenile hormone production. J Biol Chem.

[17] Weng SC, Shiao SH (2015). Frizzled 2 is a key component in the regulation of TOR signaling-mediated egg production in the mosquito *Aedes aegypti*. Insect Biochem Mol Biol.

[18] Park J-H, Attardo GM, Hansen IA, Raikhel AS (2006). GATA factor translation is the final downstream step in the amino acid/target-of-rapamycin-mediated vitellogenin gene expression in the anautogenous mosquito *Aedes aegypti*. J Biol Chem.

[19] Yu S, Wang P, Qin J, Zheng H, Wang J, Liu T (2020). *Bacillus sphaericus* exposure reduced vector competence of *Anopheles dirus* to *Plasmodium yoelii* by upregulating the Imd signaling pathway. Parasit Vectors.

[20] Kim J, Guan KL (2011). Amino acid signaling in TOR activation. Annu Rev Biochem.

[21] Umemiya-Shirafuji R, Boldbaatar D, Liao M, Battur B, Rahman MM, Kuboki T (2012). Target of rapamycin (TOR) controls vitellogenesis via activation of the S6 kinase in the fat body of the tick, *Haemaphysalis longicornis*. Int J Parasitol.

[22] Hansen IA, Attardo GM, Roy SG, Raikhel AS (2005). Target of rapamycin-dependent activation of S6 kinase is a central step in the transduction of nutritional signals during egg development in a mosquito. J Biol Chem.

[23] Shaw WR, Catteruccia F (2019). Vector biology meets disease control: using basic research to fight vector-borne diseases. Nat Microbiol.

[24] Munjuluri S, Wilkerson DA, Sooch G, Chen X, White FA, Obukhov AG (2021). Capsaicin and TRPV1 channels in the cardiovascular system: the role of inflammation. Cells.

[25] Madhumathy AP, Aivazi AA, Vijayan VA (2007). Larvicidal efficacy of *Capsicum annum* against *Anopheles stephensi* and *Culex quinquefasciatus*. J Vector Borne Dis.

[26] Antonious GF, Meyer JE, Snyder JC (2006). Toxicity and repellency of hot pepper extracts to spider mite, *Tetranychus* urticae Koch. J Environ Sci Health B.

[27] Yu S, Ji C, Zhu X, Xue J, Wang L, Wang Y (2017). Impact of *Bacillus sphaericus* exposure on *Anopheles dirus*'s fecundity and resistance development. Parasitol Res.

[28] Lu K, Wang Y, Chen X, Zhang X, Li W, Cheng Y (2018). Adipokinetic hormone receptor mediates trehalose homeostasis to promote vitellogenin uptake by oocytes in *Nilaparvata lugens*. Front Physiol.

[29] Song J, Li W, Zhao H, Zhou S (2019). Clustered miR-2, miR-13a, miR-13b and miR-71 coordinately target Notch gene to regulate oogenesis of the migratory locust *Locusta migratoria*. Insect Biochem Mol Biol.

[30] Peng L, Wang Q, Zou MM, Qin YD, Vasseur L, Chu LN (2019). CRISPR/Cas9-mediated vitellogenin receptor knockout leads to functional deficiency in the reproductive development of *Plutella xylostella*. Front Physiol.

[31] Ge L, Jiang L, Zheng S, Zhou Y, Wu Q, Liu F (2020). Frizzled 2 functions in the regulation of TOR-mediated embryonic development and fecundity in *Cyrtorhinus lividipennis* reuter. Front Physiol.

[32] Wu Z, He Q, Zeng B, Zhou H, Zhou S (2020). Juvenile hormone acts through FoxO to promote Cdc2 and Orc5 transcription for polyploidy-dependent vitellogenesis. Development..

[33] Ma L, Zhang W, Liu C, Chen L, Xu Y, Xiao H (2018). Methoprene-tolerant (Met) is indispensable for larval metamorphosis and female reproduction in the cotton bollworm *Helicoverpa armigera*. Front Physiol.

[34] Wang L, Guo Q, Levy T, Chen T, Wu X (2020). Ovarian development pattern and vitellogenesis of ridgetail white prawn *Exopalaemon carinicauda*. Cell Tissue Res.

[35] Hansen IA, Attardo GM, Park JH, Peng Q, Raikhel AS (2004). Target of rapamycin-mediated amino acid signaling in mosquito anautogeny. Proc Natl Acad Sci USA.

[36] Brandon MC, Pennington JE, Isoe J, Zamora J, Schillinger AS, Miesfeld RL (2008). TOR signaling is required for amino acid stimulation of early trypsin protein synthesis in the midgut of *Aedes aegypti* mosquitoes. Insect Biochem Mol Biol.

[37] Roy SG, Raikhel AS (2011). The small GTPase Rheb is a key component linking amino acid signaling and TOR in the nutritional pathway that controls mosquito egg development. Insect Biochem Mol Biol.

[38] Roy SG, Hansen IA, Raikhel AS (2007). Effect of insulin and 20-hydroxyecdysone in the fat body of the yellow fever mosquito *Aedes aegypti*. Insect Biochem Mol Biol.

[39] Attardo GM, Higgs S, Klingler KA, Vanlandingham DL, Raikhel AS (2003). RNA interference-mediated knockdown of a GATA factor reveals a link to anautogeny in the mosquito *Aedes aegypti*. Proc Natl Acad Sci USA.

[40] Arik AJ, Hun LV, Quicke K, Piatt M, Ziegler R, Scaraffia PY (2015). Increased Akt signaling in the mosquito fat body increases adult survivorship. FASEB J.

[41] Kawada T, Suzuki T, Takahashi M, Iwai K (1984). Gastrointestinal absorption and metabolism of capsaicin and dihydrocapsaicin in rats. Toxicol Appl Pharmacol.

[42] Chanda S, Bashir M, Babbar S, Koganti A, Bley K (2008). In vitro hepatic and skin metabolism of capsaicin. Drug Metab Dispos.

[43] Rollyson WD, Stover CA, Brown KC, Perry HE, Stevenson CD, McNees CA (2014). Bioavailability of capsaicin and its implications for drug delivery. J Control Release.

[44] Ahn SJ, Badenes-Pérez FR, Reichelt M, Svatoš A, Schneider B, Gershenzon J (2011). Metabolic detoxification of capsaicin by UDP-glycosyltransferase in three Helicoverpa species. Arch Insect Biochem Physiol.

[45] Bjedov I, Toivonen JM, Kerr F, Slack C, Jacobson J, Foley A (2010). Mechanisms of life span extension by rapamycin in the fruit fly *Drosophila melanogaster*. Cell Metab.

[46] Mason JS, Wileman T, Chapman T (2018). Lifespan extension without fertility reduction following dietary addition of the autophagy activator Torin1 in *Drosophila melanogaster*. PLoS ONE.

[47] Thoreen CC, Kang SA, Chang JW, Liu Q, Zhang J, Geo Y (2009). An ATP-competitive mammalian target of rapamycin inhibitor reveals rapamycin-resistant functions of mTORC1. J Biol Chem.

[48] Metz P, Chiramel A, Chatel-Chaix L, Alvisi G, Bankhead P, Mora-Rodriguez R (2015). Dengue virus inhibition of autophagic flux and dependency of viral replication on proteasomal degradation of the autophagy receptor p62. J Virol.

[49] Chang CF, Islam A, Liu PF, Zhan JH, Chueh PJ (2020). Capsaicin acts through tNOX (ENOX2) to induce autophagic apoptosis in p53-mutated HSC-3 cells but autophagy in p53-functional SAS oral cancer cells. Am J Cancer Res.

[50] Weng SC, Tsao PN, Shiao SH (2021). Blood glucose promotes dengue virus infection in the mosquito *Aedes aegypti*. Parasit Vectors.

[51] Hanson KK, Ressurreicao AS, Buchholz K, Prudencio M, Herman-Ornelas JD, Rebelo M (2013). Torins are potent antimalarials that block replenishment of *Plasmodium* liver stage parasitophorous vacuole membrane proteins. Proc Natl Acad Sci USA.

[52] Brennand A, Gualdron-Lopez M, Coppens I, Rigden DJ, Ginger ML, Michels PA (2011). Autophagy in parasitic protists: unique features and drug targets. Mol Biochem Parasitol.

[53] Feng Y, Chen L, Gao L, Dong L, Wen H, Song X (2021). Rapamycin inhibits pathogen transmission in mosquitoes by promoting immune activation. PLoS Pathog.

