# Supplementary material for: Capsaicin decreases fecundity in the Asian malaria vector Anopheles stephensi by inhibiting the target of rapamycin signaling pathway
Source: Parasit Vectors. 2022 Dec 12;15:458. doi: 10.1186/s13071-022-05593-0 (PMC9743593; doi:10.1186/s13071-022-05593-0)
Supplement: Supplementary file 1 — Additional file 1: Table S1. The primers used in the real-time PCR. [file 13071_2022_5593_MOESM1_ESM.docx]

Table S1. The primers used in the Real-time PCR

| Gene | Forward primer (5’-3’) | Reverse primer (5’-3’) |
| --- | --- | --- |
| *AsS7* | ATCGCTATGGTGTTCGGTTC | TCCGAGTTCATTTCCAGCTC |
| *AsAkt* | TCAATGGCGGTGAACTGTTCTTCC | CGATAGATGATGCCGTGTGAGTGG |
| *AsTOR* | CGTTCGCCTACACCAAGCATCTC | AGCCGCCTATTCTCGTCCTTCAG |
| *AsS6k* | TTACAACTCAACGGACGAGGAATGG | AACGGTTGGGCGGAATTGATCTC |
| *AsVg* | AGTCGTCCTCGTCGTCGTCTTC | GCGGTTCGGCTTCTGTTCCTC |
